# Accuracy evaluation of robot-guided laser osteotomy for dental implant bed preparation - a digital high-tech procedure

**DOI:** 10.3389/frobt.2025.1614659

**Published:** 2025-09-29

**Authors:** Florian M. Thieringer, Regina Walher, Florian S. Halbeisen, Quentin Garnier, Adrian Dragu, Bilal Msallem

**Affiliations:** 1 Clinic of Oral and Cranio-Maxillofacial Surgery, University Hospital Basel, Basel, Switzerland; 2 Department of Biomedical Engineering, Medical Additive Manufacturing Research Group (Swiss MAM), University of Basel, Basel, Switzerland; 3 Department of Clinical Research, Surgical Outcome Research Center, University Hospital Basel and University of Basel, Basel, Switzerland; 4 AOT Advanced Osteotomy Tools AG, Basel, Switzerland; 5 University Center for Orthopedics, Trauma and Plastic Surgery, Faculty of Medicine and University Hospital Carl Gustav Carus, TU Dresden, Dresden, Germany

**Keywords:** dental implant, dimensional measurement accuracy, laser ablation, oral surgery, precision medicine, robotic surgical procedures

## Abstract

**Background:**

The accuracy and reproducibility of emerging high-tech procedures for dental implant placement need continuous evaluation. This is essential to facilitate the transition from conventional surgical guides to digital planning systems. This study investigates the accuracy of implant placement using robot-guided laser technology based on cone-beam computed tomography and intraoral scanning.

**Methods:**

Twelve dental implants were placed using surgical planning software and a robot-guided laser osteotome. The procedure incorporated surface scanning and enabled implant bed preparation using a robot-guided laser.

**Results:**

The mean overall 3D offset (mean ± SD) was 2.50 ± 1.30 mm at the base and 2.80 ± 1.00 mm at the tip, with a mean angular deviation of 6.60 ± 3.10°.

**Conclusion:**

The results demonstrate a considerably greater deviation than conventional guided systems. In the context of the high demands of oral surgery, accuracy is particularly susceptible to fluctuations, some of which may stem from intermediate workflow steps, particularly due to the early development stage of the robotic system. Notably, the absence of real-time depth measurement and robot-assisted implant placement remains a significant constraint. However, future technological advances are expected to address these challenges.

## 1 Introduction

Over time, numerous techniques have been developed to enhance preoperative virtual surgical planning and the precise placement of dental implants. Implant placement methodologies may be categorized into freehand implant surgery and computer-assisted implant surgery (CAIS), which is further divided into static and dynamic approaches. Static CAIS (sCAIS) involves implants that are placed using drilling templates, whereas implementing dynamic CAIS (dCAIS) is contingent upon utilizing a real-time navigation technique (Thieringer et al., 2021; Taheri Otaghsara et al., 2023; Yu et al., 2023). The placement of dental implants requires a high degree of accuracy, not only for aesthetic and functional reasons but also for anatomical ones, such as protecting surrounding nerves, blood vessels, and teeth roots. Among these methods, guided implant placement has emerged as a widely adopted approach, with evidence to yield accurate results (Thieringer et al., 2021; Taheri Otaghsara et al., 2023; Yu et al., 2023).

Nevertheless, producing a physical guide from virtual data through 3D printing may result in inaccuracies that could affect the precision of implant positioning (Thieringer et al., 2021; Flügge et al., 2017). Recent innovations in technology and 3D simulation methods have significantly advanced the field of dentistry, particularly enhancing the safety and predictive accuracy of implant placement. A systematic review published in 2021 has highlighted several robotic initiatives in all fields of dentistry, but found that the overall quality of study designs was low, reflecting the general level of technological readiness (van Riet et al., 2021). However, dynamic surgical technologies showed considerable promise, but require further clinical validation and should be regarded as adjuncts that complement, rather than replace, surgical training and expertise (Dibart et al., 2023). Nevertheless, recent years have seen rapid advancements in the field of robotic implantology, accompanied by an increasing number of high-quality clinical studies. Preliminary findings from a narrative review suggest that robotic computer-assisted implant surgery provides sufficient accuracy and high precision in implant placement, indicating potential benefits for both clinicians and patients (Wang et al., 2024a). Currently, robotic systems are among the most widely adopted in the field of dental implantology, offering precise preoperative planning and intraoperative visual and haptic guidance, with clinical trials confirming their high placement accuracy and potential to improve long-term implant success, although there is still considerable room for further improvement (Liu et al., 2024). Robot-guided dental surgery is a highly active area of research in dental implantology, with a predominant focus being directed towards robot-guided drilling systems. While vary in design and technical implementation, these systems are unified by their reliance on mechanical drills rather than lasers. Despite this, they consistently achieve high accuracy in dental implant placement. Most of these systems have been in clinical use for several years and have demonstrated reliability across a broad range of patient cases.

In the present study, the drill was replaced with a laser capable of precise bone cutting. The key innovation lies in the integration of robotic guidance with laser osteotomy, specifically utilizing an Erbium-doped Yttrium Aluminum Garnet (Er:YAG) laser, which induces a photothermal effect leading to photoablation. Previous studies have demonstrated that laser ablation techniques utilizing Er:YAG lasers enable highly precise incisions with minimal carbonization of hard tissues and no smear layer formation (Augello et al., 2018; Moslemi et al., 2017). The mechanism of tissue ablation with Er:YAG lasers is based on the emission of a wavelength that corresponds to the absorption peak of water, allowing the energy to be absorbed by the water content in tissue. This absorption results in rapid vaporization and consequent tissue ablation. Studies have documented osteotomies reaching depths of up to 21 mm without visible thermal damage (Beltrán et al., 2020). Furthermore, the use of pulsed Er:YAG lasers preserves the structural integrity of surrounding bone tissue and has been shown to positively influence postoperative healing and the inflammatory response (Blagova et al., 2023; Xiao et al., 2023; Zeitouni et al., 2017). Compared to conventional drilling tools, laser-based osteotomy produces less debris and fewer blood cells at the implant bed, factors that, when excessive, may impair the osseointegration process (Semez et al., 2018). Evidence also suggests that Er:YAG lasers better preserve trabecular bone architecture at the osteotomy margins, making it potentially more biologically favorable than traditional rotary instruments. In contrast, conventional drilling is associated with trabecular collapse and thermal damage, which can delay healing by restricting cellular migration and inducing localized necrosis (Zeitouni et al., 2017). Laser osteotomes operate without mechanical contact, providing a non-contact, blood-, heat-, and vibration-reduced alternative to conventional methods, potentially enhancing postoperative bone healing (Ebeling et al., 2023). Crucial laser parameters, such as pulse duration and frequency, have been identified as decisive factors for the procedure (Moslemi et al., 2017; Xiao et al., 2023). However, technical constraints associated with controlling the depth remain a significant limitation (Xiao et al., 2023; Ureel et al., 2021). Therefore, the present study evaluates a system that combines advanced robotic guidance, eliminating the need for surgical guides or impressions, with laser osteotomy as a biologically favorable osteotomy method, while also allowing compatibility with future technological developments.

**FIGURE 1:**
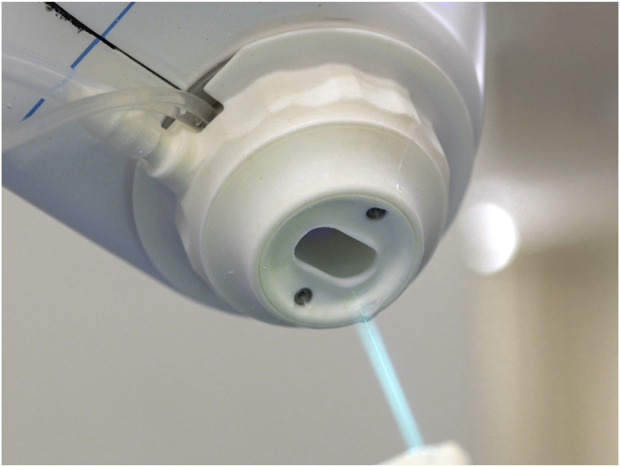
Laser beam emitted from a robot-guided laser osteotome.

The Cold Ablation Robot-Guided Laser Osteotome (CARLO®), an autonomously acting robotic arm equipped with a laser head at the end, is already being used in clinical practice (Ebeling et al., 2023; Ureel et al., 2021; Holzinger et al., 2021; Ettl et al., 2023; Berg et al., 2019; Köhnke et al., 2024). The CARLO® system enables highly precise osteotomies through the integration of robotic guidance and robotic laser osteotomy (Figure 1). The employment of robot-guided laser osteotomes within the operating theatre has been demonstrated to exhibit both ergonomic and safety benefits (Köhnke et al., 2024; Baek et al., 2015). Accuracy in this system is ensured through the integration of optical tracking, high-precision robotic actuation, and calibrated registration protocols. The system uses a stereoscopic infrared optical tracking system to continuously monitor both the laser applicator and a rigid reference array affixed directly to the patient’s bone via a bone screw, ensuring rigid-body coupling. Registration is achieved through point-based surface matching using anatomical landmarks, with transformation matrices computed to align the physical coordinate system to the preoperative 3D imaging data. Registration fidelity is quantitatively assessed and consistently maintained below 1.0 mm. The laser applicator is mounted on a seven-degree-of-freedom collaborative robotic manipulator, offering sub-millimetric positional repeatability (± 0.15 mm) and angular repeatability below 0.01 rad. The robotic arm follows a predefined osteotomy trajectory based on the virtual surgical plan, with positional feedback ensuring consistent adherence to the planned path. Any deviation due to intraoperative movement or marker displacement is immediately detected by the navigation system, which triggers a procedural halt until re-registration is completed. This closed-loop, error-sensitive navigation ensures spatial fidelity is maintained throughout the osteotomy. Currently, depth control is manually supervised, with surgeons relying on real-time visual feedback, such as displayed laser trajectory, ablation depth and ablation endpoint status, as well as acoustic changes during bone cutting. The current system does not include automatic cut-off functionality based on tissue transition detection. The maximum cutting depth is 20 mm, with a total width of 1.5 mm.

**FIGURE 2:**
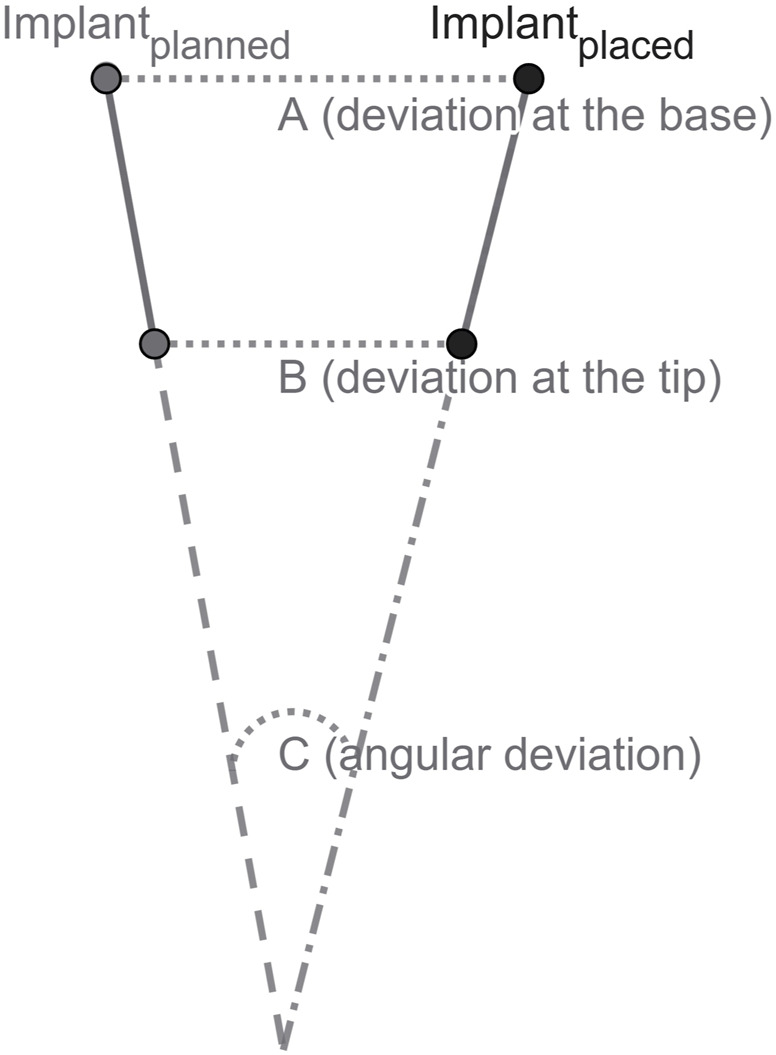
Schematic illustration of implant position: **(A)** deviation at the base; **(B)** deviation at the tip; and **(C)** angular deviation.

The aim of this study is to assess the accuracy of robot-guided laser osteotomy for dental implant bed preparation and the subsequent implant positioning using an animal cadaver model. The primary objective is to evaluate the precise transfer of a digital treatment plan to a cadaver mandible, ensuring optimal implant placement. High accuracy is defined as the minimal deviation in measurements at the base, tip, and angle when comparing the planned position in the virtual treatment plan to the actual implant position (Figure 2).

This study encompasses the acquisition of digital data, virtual implant planning, guided implant bed preparation using an autonomous surgical laser robot, and the subsequent digital evaluation of outcomes. The workflow applied in this study was defined based on established protocols as described in the literature (Thieringer et al., 2021; Joda et al., 2017; Franchina et al., 2020). Additionally, the study provides an evaluation of the robotic computer-assisted implant surgery workflow in terms of feasibility, safety, and reliability.

## 2 Materials and methods

This study assesses the accuracy of robot-guided implantation utilizing robotic laser technology for implant bed preparation (*n* = 12) within a designated edentulous area of the mandible in animal cadavers.

**FIGURE 3:**
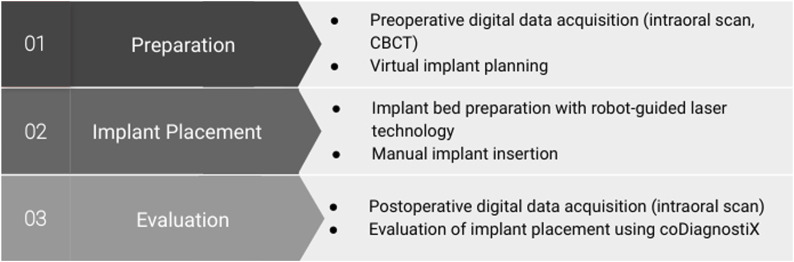
Overview of the study protocol.

Digital data were obtained using a cone-beam computed tomography (CBCT) and an intraoral scanner. These datasets were imported into the planning software coDiagnostiX® v. 10.6 (Dental Wings Inc., Montreal, Canada), where they were merged into a model for virtual planning. Following robot-guided surgery and implant placement, a second intraoral scan of the surface with scan bodies was acquired and superimposed on the preoperative model. The discrepancy between the planned position and the actual implant position was analyzed using a treatment evaluation module of coDiagnostiX®. Descriptive statistics were employed to evaluate the results. A detailed description of the different study modules is provided below (Figure 3).

### 2.1 Preparation

#### 2.1.1 Preoperative digital data acquisition

Two lower sheep jaws were obtained from a local butcher’s abattoir waste. CBCT scans were performed using a CS 9300 (Carestream Dental LLC, Atlanta, GA, United States) with the following settings: 90 kV, 5.6 mA, 200 μm voxel size, 8 × 8 cm field of view. The acquired data were exported into a Digital Imaging and Communications in Medicine (DICOM) file. Additionally, the surface areas of the jaws were captured utilizing an intraoral scanner TRIOS® (3Shape A/S, Copenhagen, Denmark) to ascertain the intraoral status of the surface.

#### 2.1.2 Virtual implant planning

**FIGURE 4:**
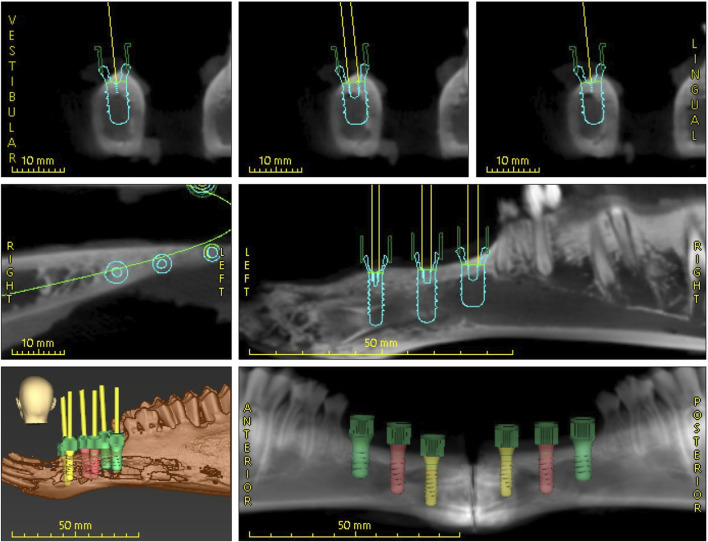
Digital dental implant planning performed using coDiagnostiX® software.

The intraoral scan and the CBCT images were imported into coDiagnostiX® (Dental Wings Inc., Montreal, Canada) and merged to create a virtual model. The software, certified for clinical use, facilitated the planning process. An edentulous area was selected for implant placement, and three pairs of Roxolid® SLA® implants (Straumann AG, Basel, Switzerland) were virtually positioned with the following specifications: tissue level with diameters of 3.3 mm and 4.1 mm, both with a length of 10 mm, and implants with a diameter of 4.8 mm and a length of 8 mm. Given the anatomical differences conditions between sheep and human jaws, the course of the mandibular nerve was not considered in the planning process. A Standard Tessellation Language (STL) file of the model was generated. The results thereof are illustrated below (Figure 4).

### 2.2 Implant placement

#### 2.2.1 Implant bed preparation with robot-guided laser technology

The STL-planning file from coDiagnostiX® was transferred to the CARLO® primo + software v. 2.0.2 (AOT AG, Basel, Switzerland). A patient marker was affixed to the mandible to enable visual tracking. Initial landmark registration was performed using a round pointer, followed by a surface-matched point cloud registration. The final registration had achieved a deviance of less than 1.0 mm. During the operation, the navigation system continuously monitored the position of the lower jaw using three reflective markers. Subsequently, six implant beds were prepared by laser for each jaw. The pulsed Er:YAG laser emits a wavelength of 2,940 nm. The following parameters were used.

- Pulse frequency: 10 Hz
- Spot size: 0.9 mm
- Optical Power: 6.8 W

#### 2.2.2 Manual implant insertion

**FIGURE 5:**
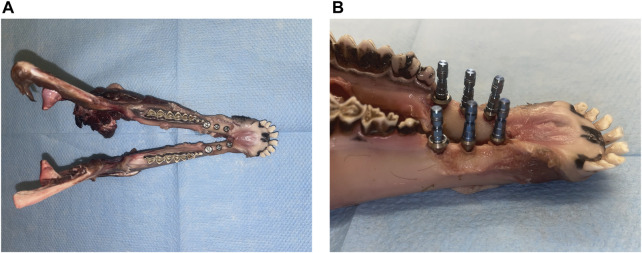
Views of the sheep lower jaw after implant insertion: **(A)** superior view and **(B)** lateral view.

Following the treatment plan, six implants were manually placed in each of the lower jaws (Figure 5). The implants included three pairs of Roxolid® SLA® implants with following specifications: ∅ 3.3 mm/10 mm, ∅ 4.1 mm/10 mm, and ∅ 4.8 mm/8 mm.

### 2.3 Evaluation

#### 2.3.1 Postoperative digital data acquisition

Postoperative dental surface scans of each mandible were conducted for all implants using scan bodies (Straumann AG, Basel, Switzerland) and the same intraoral scanner employed during the initial data acquisition.

#### 2.3.2 Evaluation of implant placement using coDiagnostiX®

**FIGURE 6:**
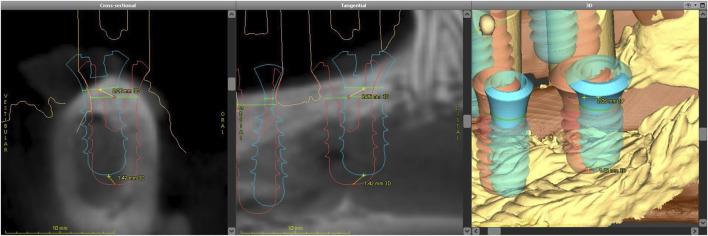
Dental implant alignment illustrating preoperative positioning (red) and postoperative positioning (blue).

Postoperative data were imported into the coDiagnostiX® software and analyzed using the treatment evaluation module. The preoperative 3D models were superimposed onto the postoperative surface scans to confirm anatomical structures and facilitate postoperative assessment. Implant alignment for each tooth position was determined through a semi-automatic process, as demonstrated. This process was systematically repeated for all implants. The treatment evaluation module provided a visual map and generated a spreadsheet to calculate the difference between the planned and actual base, tip, and angle values (Figure 6).

### 2.4 Statistics

Statistical analyses were performed using the R statistical software package (v. 4.2.2, The R Foundation for Statistical Computing, Vienna, Austria). Descriptive statistics, including mean, standard deviation (SD), median, and interquartile range (IQR), were used to evaluate the differences between the actual implant placements and the virtual planned implant positions. This study recorded differences in angle, 3D displacement, and dimensions between the base and tip.

## 3 Results

A summary of the relevant characteristics and statistical results of this study are presented in Table 1. The overall mean 3D deviation at the base (entry point) was 2.50 ± 1.30 mm, while at the tip (apex point) it was 2.80 ± 1.00 mm. The mean angular deviation was 6.60 ± 3.10°.

**TABLE 1:** Summary of angular, base, and tip deviation values.

Characteristic	*n* ^a^	Median (IQR ^b^)	Mean (SD ^c^)
Angular Deviation (°)			
Deviation (°)	12	6.2 (5.5 to 8.7)	6.6 (3.1)
Base Deviation (mm)			
3D offset - Base	12	2.4 (1.7 to 3.4)	2.5 (1.3)
Distal - Base	12	−0.4 (−1.6 to 1.0)	0.0 (2.1)
Vestibular - Base	12	−0.4 (−0.8 to 0.6)	0.1 (1.5)
Apical - Base	12	0.3 (−1.1 to 0.8)	−0.1 (1.3)
Tip Deviation (mm)			
3D offset - Tip	12	2.6 (2.0 to 3.7)	2.8 (1.0)
Distal - Tip	12	−0.9 (−1.6 to 0.6)	−0.3 (2.4)
Vestibular - Tip	12	−1.2 (−1.4 to −0.1)	−0.6 (1.4)
Apical - Tip	12	0.4 (−1.0 to 0.9)	−0.1 (1.3)

^a^ Number of replicas.

^b^ Interquartile range.

^c^ Standard deviation.

A comparison between the planned and inserted implant positions revealed a mean deviation at the implant base of 0.00 ± 2.10 mm in the distal region and 0.10 ± 1.50 mm in the vestibular region. In contrast, the largest discrepancies were observed at the implant tip in the distal region, with a mean deviation of −0.30 ± 2.40 mm, and in the vestibular region, with a mean deviation of −0.60 ± 1.40 mm. The mean deviation, considering both the apical base and the apical tip measurements, was −0.10 ± 1.30 mm for both.

**FIGURE 7:**
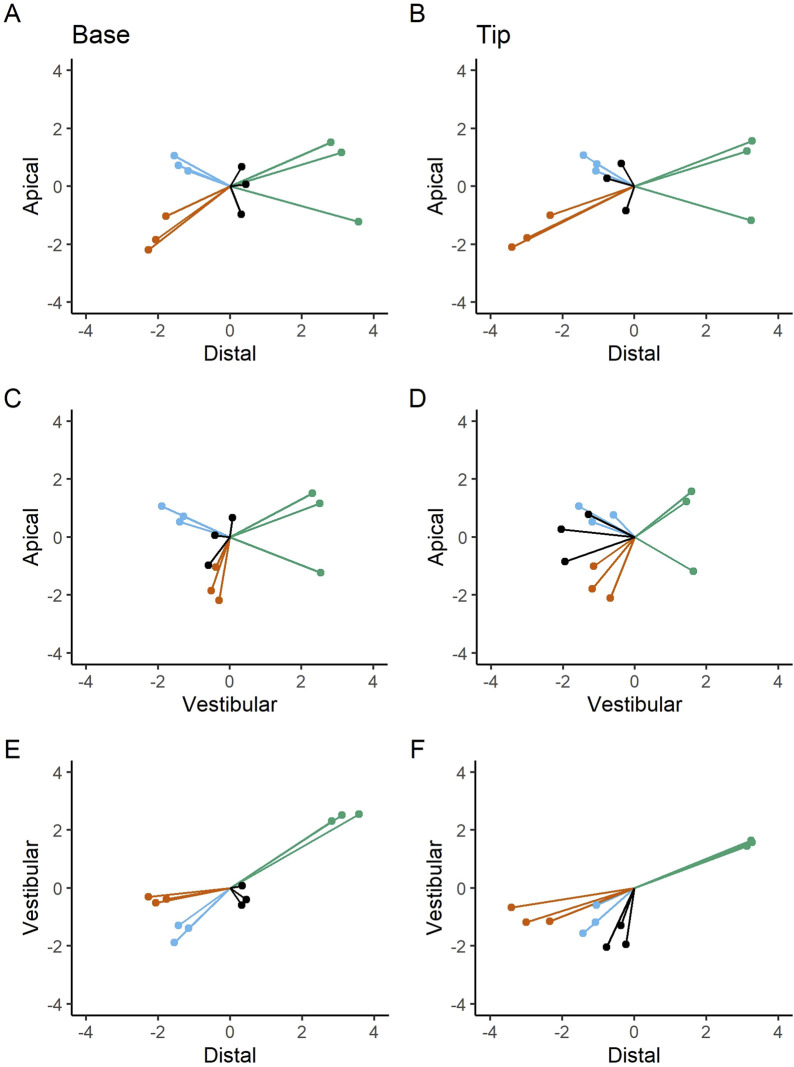
Apical-distal deviations at the base **(A)** and tip **(B)**; apical-vestibular deviations at the base **(C)** and tip **(D)**; vestibular-distal deviations at the base **(E)** and tip **(F)**. Bilateral deviation measurements are presented in millimeters (mm). Dots and lines represent the extent and orientation of the deviations.

The subsequent figure depicts the deviations observed at the implant base and tip across the apical-distal, apical-vestibular, and vestibular-distal directions (Figure 7). The current data set indicates that deviations tend to occur in clusters. The most pronounced discrepancies were observed in the apical-distal versus apical-vestibular direction at the implant base (A) and tip (B), with additional notable differences in the apical-vestibular versus vestibular-distal direction at the implant base (E) and tip (F). The results suggest that the measured deviation values are not entirely independent. For example, when comparing distal and vestibular values, a high distal value leads to a high vestibular value, and *vice versa*. In contrast, the apical values appear to be distributed rather randomly, with no discernible pattern.

**FIGURE 8:**
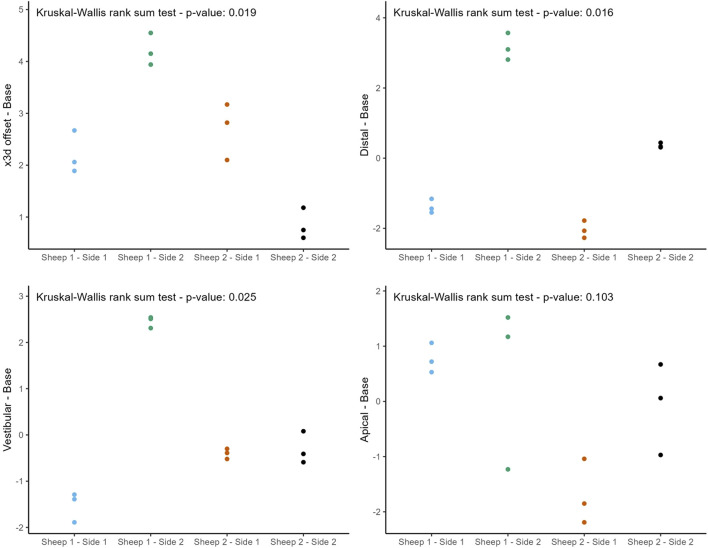
Individual deviations at the base categorized by jaw side.

**FIGURE 9:**
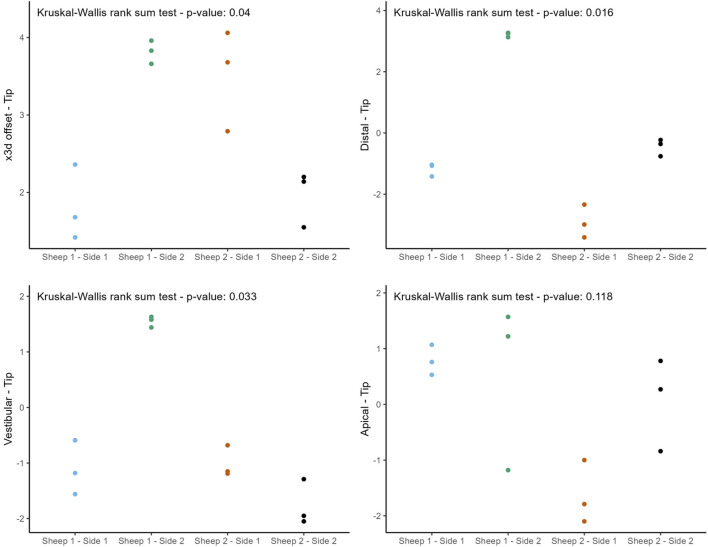
Individual deviations at the tip categorized by jaw side.

The following figures (Figures 8, 9) provide a more nuanced understanding of the differences between the various implant positions. Notably, the deviations observed on both sides of the jaws are consistently grouped and close to each other. This observation could strengthen the possibility of a systemically influenced deviation pattern per side.

## 4 Discussion

This study provides a comprehensive and systematic analysis of robot-guided laser ablation technology in dental implantology, with a particular focus on the accuracy of autonomous robot-assisted procedures utilizing laser technology for implant bed preparation based within a partially digital workflow. The overall mean 3D deviation at the base was 2.50 ± 1.30 mm, while at the tip it was 2.80 ± 1.00 mm. The mean angular deviation was 6.60 ± 3.10°. The International Team for Implantology’s fifth Consensus Report recommends an additional 2 mm when planning implant placement near vital anatomical structures or adjacent implants, favors fully guided implant insertion over guided implant bed preparation alone, and advises intraoperative periapical radiographs in borderline cases to enhance safety (Tahmaseb et al., 2014). In the present study, the observed deviations exceed this recommended threshold, potentially increasing the risk of injury to adjacent teeth and neurovascular structures. These findings suggest that the application of this specific technology in dental implant placement may result in considerable deviations from the planned positions. Notably, the results indicate similar levels of deviation at both the base and tip. A detailed examination of Figures 6, 8, 9 reveals a relatively consistent implantation offset across all implants on each side, which may indicate the presence of a systemic error. This observation supports the assumption of an application-related error rather than a hardware malfunction. It is important to note that only the implant bed was prepared using a laser-based technique, while several steps in the workflow were still performed manually. These manual interventions may have contributed to the observed inaccuracies.

From a technical perspective, the accuracy can be affected by various aspects of the digital workflow. Literature indicates that segmentation and registration are crucial factors in software-based planning, with imaging artifacts and data registration errors impacting the planning phase (Block, 2023; Li et al., 2023; Wei et al., 2023). Block and Flügge et al. identified discrepancies arising from data acquisition, particularly during the registration process between surface scans and CBCT data (Flügge et al., 2017; Block, 2023). Furthermore, discrepancies may arise during the transfer of the virtual surgical plan to the CARLO® system, as only a subset of data files is currently supported, necessitating intermediate steps, particularly for the depth control. In addition, low bone density in the anterior region of the jaw where the implants were to be placed during digital planning with coDiagnostiX® presented a challenge in determining the border of the alveolar crest. As outlined by Block, such challenges can contribute to reduced accuracy in implant placement (Block, 2023). Additional inaccuracies and artifacts may have been introduced during implant bed preparation due to the manual landmark and surface registration process with a round-tipped pointer. Although the software provided sufficient values after the registration check without indicating errors, it is reasonable to assume that the use of a sharp-tipped pointer could have yielded more precise measurements and reduced the likelihood of deviations. Li et al. and Ureel et al. have emphasized that the type and fixation of the positioning marker are crucial for accurate registration (Li et al., 2023; Ureel et al., 2021). An optical surface scanner is currently under development to enhance the registration process. The trabecular structure of the medullary cavity poses another challenge for both automatic and manual depth measurement. A precise automated measurement mechanism for depth control during the osteotomy process could potentially improve accuracy. At the time of this study, the robotic laser device lacked automatic depth control, which remains under development. Hamidi et al. contributed to the development of an integrated optical feedback system aimed at enhancing depth monitoring and control for laser-induced osteotomies (Hamidi et al., 2023).

To improve accuracy, tooth-supported guides are typically employed to enhance the transfer of planned implant positions. A study by Derksen et al. investigated the accuracy of implant placement using coDiagnostiX® generated surgical guides, reporting a mean deviation of 0.75 ± 0.34 mm at the base, 1.06 ± 0.44 mm at the tip, and a mean angular deviation of 2.72 ± 1.42° (Derksen et al., 2019). These values suggest that guided surgery using tooth-supported surgical guides created in a digital workflow results in less deviations compared to the present study. Previous studies have demonstrated the high accuracy of osteotomies performed using the CARLO® system, with a high degree of precision between the planned and executed osteotomies (Holzinger et al., 2021; Ettl et al., 2023; Msallem et al., 2024). However, in dentistry, even minor deviations of approximately 1 mm are clinically significant (Block, 2023). Precise implant positioning within a sub-millimeter range is essential for successful placement, whereas osteotomies allow for slight deviations in the millimeter range. Monje et al. recommend maintaining a minimum distance of 1.5 mm from the buccal bone to ensure implant stability (Monje et al., 2019). Taheri Otaghsara et al. found that implant placement accuracy depends on the implant site and that dCAIS shows a more significant deviation in the mesial direction (Taheri Otaghsara et al., 2023). A study by Li et al. reported significant results for base deviation of 0.67 ± 0.37 mm, tip deviation of 0.69 ± 0.37 mm, and angular deviation of 1.27 ± 0.59° in autonomous robotic surgery (Li et al., 2023). The study used robotic computer-assisted implant surgery for implant placement in fully edentulous patients, with implant bed preparation performed via robotic drilling. Another study demonstrated that human-robot interaction influences the efficiency of dental implant surgery, with passive robot-assisted systems showing greater implant placement deviations (Xu et al., 2023). Yu et al. conducted a review on the accuracy of different implant planning and placement methods. Their review focused on measuring the accuracy of implant placement by base deviation, tip deviation, and angular deviation. The findings of Yu et al. confirmed that implant placement using dCAIS approaches is more accurate than freehand techniques and observed less angular deviation compared to sCAIS (Yu et al., 2023).

A systematic review and meta-analysis of clinical studies published between August 2014 and October 2024 confirmed that robotic computer-assisted implant surgery achieves high accuracy, but large-scale, multicenter randomized controlled trials are needed to compare it with alternative techniques (Luo et al., 2025). The Yomi® (Neocis Inc., Miami, FL, US), the first FDA-cleared robot-assisted dental surgical system, demonstrated superior accuracy in a clinical series of 273 implants, with a mean base deviation of 1.10 ± 0.69 mm, tip deviation of 1.12 ± 0.69 mm, and angular deviation of 1.42 ± 1.53°, outperforming freehand, static, and dynamic computer-guided methods (Neugarten, 2024). Clinical trials of the Remebot® (Baihui Weikang Technology Co., Ltd., Beijing, China) also demonstrated high accuracy and safety, with a median base deviation of 0.62 mm (0.46–1.00 mm), tip deviation of 0.62 mm (0.49–1.01 mm), and angular deviation of 1.16° (0.69–1.69°) (Liu et al., 2025). The Yakebot® (Yake Wisdom (Beijing) Technology Co., Ltd., Beijing, China) demonstrated higher accuracy, safety, and flexibility as well, compared to static templates, achieving a mean base deviation of 0.65 ± 0.25 mm, tip deviation of 0.65 ± 0.22 mm, and angular deviation of 1.43 ± 1.18° (Wang et al., 2024b). Similarly, the Langyue dental surgical robot (Shecheng Co., Ltd., Shanghai, China) demonstrated high accuracy in an edentulous arch, with a mean base deviation of 0.53 ± 0.17 mm, tip deviation of 0.58 ± 0.17 mm, and angular deviation of 0.77 ± 0.26° (Qiao et al., 2023). The THETA system (Hangzhou Jianjia Robot Co., Ltd., Hangzhou, China) outperformed a dynamic navigation system, demonstrating a mean base deviation of 0.58 ± 0.31 mm, tip deviation of 0.69 ± 0.28 mm, and angular deviation of 1.08 ± 0.66° (Chen et al., 2023). A recent randomized controlled trial with a 6-month follow-up demonstrated that the THETA system (Hangzhou Jianjia Robot Co., Ltd., Hangzhou, China) significantly outperforms the freehand technique in terms of accuracy, showing mean base deviations of 0.70 ± 0.11 mm, tip deviations of 0.70 ± 0.12 mm, and an angular deviation of 1.09 ± 0.67°, compared to 1.24 ± 0.59 mm, 2.13 ± 1.26 mm, and 7.43 ± 6.12°, respectively, in the freehand group (Chen et al., 2025). Overall, all current robotic systems in dental implantology demonstrate high accuracy and safe handling, with virtually all employing rotary drills for implant bed preparation.

The findings of this study are subject to several limitations. A primary limitation is the relatively small sample size, which constrains the statistical power and generalizability of the results. The limited number of implants used was primarily due to the substantial financial costs associated with the experimental setup, which required a significant amount of labor. This was largely because the CARLO® system is still in the development phase and resource availability was limited. Given the considerable deviations observed, one potential source of error may be operator-related, as several steps in the workflow were still performed manually, such as landmark registration using a round-tipped pointer. The surface registration method employing a surface scanner might have resulted in improved accuracy. Another limitation of the study is the lack of guided implant insertion, which is standard in fully guided workflows. Implant placement was performed manually due to the absence of an integrated robotic positioning device, which may have introduced further inaccuracies, possibly caused by increased insertion force. The potential for greater accuracy may be achieved if future developments allow the robotic arm to insert the implants autonomously. Also, the anatomical characteristics of the sheep jaw present further challenges. The sheep mandible is characterized by a thicker cortical bone layer and relatively sparse trabecular spongiosa, which may increase the difficulty of achieving accurate depth control during implant bed preparation. In contrast, the human mandible typically features thinner cortical bone and a denser trabecular structure, offering different mechanical properties. Sheep exhibit a distinctive mandibular anatomy, characterized by a fibrous connective tissue connection in the anterior region at the junction between the right and left jaws. This anatomical feature introduces a degree of instability, particularly due to the elongated jaws, which generates large lever forces, posing challenges for both optical registration and radiological assessment. The findings illustrated in Figures 8, 9 further support these observations. These anatomical and structural disparities highlight the limitations of the sheep model and emphasize the need for further validation through human cadaveric studies. Furthermore, this study did not account for the limitations on mouth opening due to soft tissue, such as the lips and cheek, which would be particularly relevant when working in the distal region of the mouth. In such cases, the perioral soft tissues would represent a limiting factor for mouth opening, thereby restricting the handling of the currently still relatively large laser head.

However, future technological advancements are to overcome current limitations. Innovations such as surface scanner–based registration, real-time depth control, miniaturization through more compact robotic devices, and integrated intraoperative imaging will improve both precision and usability. This study has demonstrated that this novel technology remains in the early stages of development. While the technology has shown promising outcomes in surgical applications like osteotomies, it is not yet fully optimized for dental implantology. The present findings confirm the feasibility of the proposed workflow. However, more sophisticated technologies are currently available. Looking ahead, this system has the potential to offer advantages beyond laser-based implant bed preparation. These include the elimination of physical drill guides, drill system sterilization costs, improved precision in robot-guided implant surgery and the utilization of the biological benefits of laser treatment. Unlike conventional drills, lasers operate independently, remain perpetually sharp and can be deactivated immediately, offering a sterile and efficient alternative. In the longer term, fully autonomous implant placement through robot-guided positioning is a realistic objective. Furthermore, the integration of artificial intelligence and machine learning holds significant promise for future applications, with the potential to enable real-time anomaly detection and adaptive intraoperative guidance, ultimately enhancing surgical precision and outcomes. The CARLO® system is not limited to dentistry, rather, its innovative use of a laser in place of a conventional drill positions it for broader, cross-disciplinary applications, particularly within large, multidisciplinary care centers. Cold ablation robot-guided laser osteotomy is gaining momentum in orthopedic surgery, particularly in procedures involving the hand, wrist, and forearm, where it offers the advantage of customizable cutting patterns that promote primary bone stability and may reduce the reliance on hardware for osteosynthesis (Honigmann et al., 2022). In a multicenter study, the CARLO® system demonstrated excellent feasibility, safety, and precision in performing linear midface osteotomies, with a 100% technical success rate and deviations of less than 2 mm in 96% of cases, while also reducing invasiveness and enhancing simplicity, safety, reliability, and accuracy (Köhnke et al., 2024). A proof-of-principle study further validated the system’s ability to create highly precise channels in the skull bone with accuracy sufficient to guide biopsy needle insertion without the need for additional navigational aids, an important step toward less invasive neurosurgical interventions (Ha et al., 2022). Moreover, an *in vitro* study introduced a novel approach to fronto-orbital advancement by combining robot-guided laser osteotomy with 3D-printed, patient-specific implants, eliminating the need for traditional surgical templates while enabling highly precise osteotomies and potentially enhancing surgical efficiency, particularly in pediatric craniofacial surgery (Maintz et al., 2025).

It is expected that challenges will arise when developing innovative technologies. However, these limitations are manageable and can be systematically overcome with further research and technological refinement.

## 5 Conclusion

This study demonstrates the feasibility of a partially digital workflow for dental implantology, encompassing data acquisition of an edentulous jaw, implant planning, and preparation of the implant bed with an autonomous robot-guided laser. The overall mean 3D deviation (mean ± SD) was 2.50 ± 1.30 mm at the implant base and 2.80 ± 1.00 mm at the tip, with a mean angular deviation of 6.60 ± 3.10°. A considerable deviation at the implant base, as well as the implant tip and angulation were observed. However, the consistent implantation offset on both sides suggests the presence of a potential systemic error. This finding indicates that an application error is more likely to be the source of the issue. Further advancements in robotic guidance, laser technology (such as surface scanner–based registration), real-time depth control, miniaturized robotic devices, and integrated intraoperative imaging are expected to overcome current limitations and significantly enhance accuracy in future applications. Although the accuracy of this robotic system is currently inferior to that of other systems currently available on the market. The proposed workflow eliminates the need for implant guides, specialized drills or extensive bone exposure, potentially reducing treatment time and improving overall quality.

This study highlights key areas for improvement that should be addressed in future procedural planning. Specifically, further investigations are required to integrate real-time depth control and facilitate progression toward fully autonomous implant placement. Moreover, the limitations of the current animal model underscore the need for validation in human cadaveric studies.

## Data Availability

The original contributions presented in the study are included in the article/supplementary material, further inquiries can be directed to the corresponding author.
